# A phylogenetic examination of the primary anthocyanin production pathway of the Plantae

**DOI:** 10.1186/1999-3110-55-10

**Published:** 2014-01-25

**Authors:** James J Campanella, John V Smalley, Maureen E Dempsey

**Affiliations:** 1 Department of Biology and Molecular Biology, Montclair State University, 1 Normal Avenue, Montclair, New Jersey 07043 USA; 2 Department of Biology and Horticulture, Bergen Community College, 400 Paramus Road, Paramus, New Jersey 07652 USA

**Keywords:** Molecular evolution, Anthocyanin, Dihydroflavonol reductase, Anthocyanin synthase, Flavonoid hydroxylase, Glucosyltransferase

## Abstract

**Background:**

Anthocyanin pigments aid in reproduction and provide ultraviolet protection to land plants. We have examined the phylogenetic relationships among the five primary enzymes responsible for producing anthocyanin pigment in its three major forms. Dihydroflavonol 4-reductase (DFR), anthocyanidin synthase (ANS), Flavonoid 3’glucosyltransferase (F3GT), flavonoid 3’hydroxylase (F3’H), and flavonoid 3’5’ hydroxylase (F3’5’H) are responsible for the final steps in anthocyanin pigment production.

**Results:**

We were interested in how conserved the anthocyanin pathway genes may be among land plants, and evolutionarily how far back into the plant lineage anthocyanin production may be traced. The DFR, ANS, F3GT, and F3’H genes date back 450 million years to the first land plants. Mosses, spike mosses, and ferns express these four products, although there is no evidence of sequence orthologues for these genes in algae. Additionally, F3’5’H is not evident in organisms that predated gymnosperms.

**Conclusion:**

Our findings support the hypothesis that “blue” anthocyanin pigments did not evolve until 300-350 mya along with the gymnosperms, although the “red” anthocyanin pigments may be as ancient as the mosses (~450 mya).

**Electronic supplementary material:**

The online version of this article (doi:10.1186/1999-3110-55-10) contains supplementary material, which is available to authorized users.

## Background

The anthocyanin flavonoid pigments are ancient secondary metabolites of land plants that can be biochemically detected in species as ancient as mosses (Bendz 1961). Anthocyanins have two major roles in plant physiology. First, they provide visual cues to animals to help induce pollination and seed spread. Therefore, the anthocyanin family of pigments is responsible for most red, orange, pink, purple and blue flowers and fruits observed in nature. The second function of anthocyanins is as a light energy absorbance and dispersal system. These pigments can diminish photo-oxidative injury in leaves, both by protecting chloroplasts from excess high-energy quanta and by scavenging reactive oxygen species. Anthocyanins defend plants against ultraviolet radiation damage by absorbing in the 280–320 nm wavelengths (Takahashi et al. 1991; Li et al. 1993). Additionally, these pigments can absorb wavelengths between 500 and 550 nm and may also reflect across a wide region near 600–640 nm (Gausman 1983; Neill and Gould 2003).

**Figure 1:**
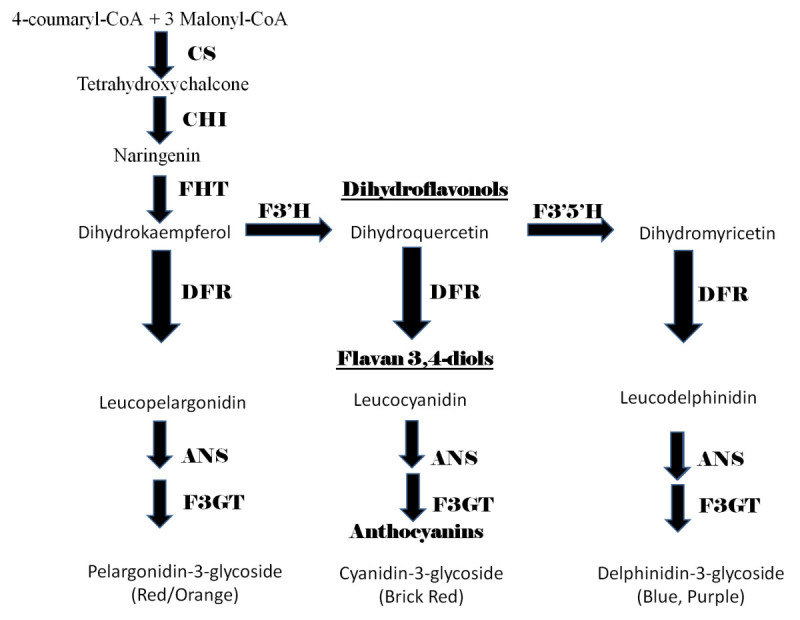
**Anthocyanin biosynthetic pathways for the three major pigment types.** CS = Chalcone synthase, CHI = Chalcone isomerase, FHT = Flavanone 3-hydroxylase, DFR = Dihydroflavonol 4-reductase, ANS = Anthocyanidin synthase, F3GT = Flavonoid 3′-glucosyltransferase, F3′H = Flavonoid 3′-hydroxylase, F3′5′H = Flavonoid 3′5′-hydroxylase.

There are three major anthocyanin pigment types found in terrestrial plants: delphinidin-3-glycoside (blue/purple), cyanidin-3-glycoside (brick red), and pelargonidin-3-glycoside (orange/red) (Grotewold 2006) (Figure 1). The production of the pigment type varies from species to species depending upon which primary enzymatic components are available. For example, irises and delphinium primarily produce the blue/purple delphinidin pigment, while geraniums primarily produce the bright red pelargonidin pigment (Grotewold 2006). The anthocyanin chromophores are generally present in the plant cell vacuoles. Anthocyanins are also present in the vegetative tissues, including leaves, stems, and even roots. They are discernible in the leaves of autumn foliage and in developing shoots. The actual color variation that is visible in tissues is also dependent on cellular pH.

The three major anthocyanin pigments are the end products of the complex flavonoid pathway which begins with the condensation of one molecule of 4-coumaroyl-CoA and three molecules of malonyl-CoA by Chalcone synthase (CS) (Buchanan et al. 2000) (Figure 1). Tetrahydroxychalcone is produced in this pathway by CS and is then converted to naringenin by chalcone isomerase. Naringenin is the flavone precursor for the dihydroflavonol dihydrokaempferol (DHK). The anthocyanin pathway itself uses DHK as its primary precursor (Grotewold 2006) (Figure 1). Dihydrokaempferol may be either converted into dihydroquercetin (precursor of the dark red/mauve pigment pathway) by flavonoid 3′hydroxylase (F3′H) or into leucopelargonidin (precursor of the red/orange pigment pathway) by dihydroflavonol 4-reductase (DFR) (Figure 1). Leucopelargonidin is then converted into the red pigment pelargonidin by anthocyanidin synthase (ANS). Dihydroquercetin may itself follow two different pathways, either being converted into leucocyanidin, a flavan 3,4-diol, on the way to being the brick red anthocyanin cyanidin, or being additionally hydroxylated by flavonoid 3′5′hydroxylase (F3′5′H) to give dihydromyricetin, the precursor for the blue, delphinidin anthocyanins (Figure 1). Plants producing the bright red pelargonidin-pigmented flowers do not necessarily need to make the enzymes F3′H or F3′5′H enzymes. Cyanidin-producing plants require expression of F3′H, and delphinidin-producing plants must have F3′5′H expression to yield purple or blue flowers. The final step for conversion of the major leucoanthocyanins to anthocyanins is first anthocyanidin production by ANS followed by glucosylation by flavonoid 3′glucosyltransferase (F3GT), also known as UDP glucose-flavonoid 3-o-glucosyl transferase.

Biochemical studies suggest that all higher plants produce anthocyanins. In addition to about half of moss species (Markham 1988; Mues 2002), anthocyanins are detectable in ferns (Harborne 1965), as well as gymnosperms and angiosperms. Surveys of ferns have shown that 80 to 90% of anthocyanins in young leaves are the brick-red colored, cyanidin-3-glycoside (Harborne 1967; Harborne 1979). Furthermore, this pigment family can be identified in angiosperms, such as sea grasses, that have returned to an aquatic habitat after initially evolving on land (Trocine et al. 1981).

Limited evolutionary studies have been performed on the anthocyanin biosynthetic pathway across a range of species. Rausher et al. (1999) examined the evolutionary rate variation among genes in the pathway using the species maize, morning glory, and snapdragon. They found the “upstream” (chalcone synthase, and chalcone flavanone isomerase) anthocyanin pathway genes have evolved more slowly than the “downstream” genes (DFR, ANS, F3′H, F3′5′H, and F3GT) in all three species. Lu and Rausher (2003) further determined upon examining a larger number of six species that chalcone synthase evolved more slowly than the furthest downstream anthocyanin elements ANS and F3GT. We have been able to find no reports in the literature of the delphinidum pigment being produced in gymnosperms.

Caputi et al. (2012) performed a phylogenetic reconstruction of the UDP-glycosyltransferases (UGTs). The research examined over 1500 putative UGTs in 12 plant genomes based on the highly conserved PSPG amino acid motif. Their data suggested that the UGT family increased in size during the transition from algae (1 UGT) to vascular plants (74) to higher plants (85–241 UGTs). Caputi et al. (2012) were able to detect UGTs in moss and algae, but they identified none as being in the anthocyanin-related GT clade.

The purpose of this present study is to examine the molecular evolution, phylogenomics, and relationships among the five major enzymes (DFR, ANS, F3′H, F3′5′H, and F3GT) defined as the “downstream” biochemical components that produce anthocyanin pigments across the whole range of species within Plantae. Characterization of such relationships will clarify how production of these pigments has evolved in terrestrial plants along with how the functional genomics of the enzymes in these pathways have changed over evolutionary time. Additionally, it is our intent to determine whether the interspecific relationships of these molecules reflect recognized evolutionary hypotheses in the Plantae.

## Methods

### Sources of sequence data

All sequences were obtained from either the Gene Index Project site at Harvard (http://compbio.dfci.harvard.edu/tgi/) or GenBank (http://www.ncbi.nlm.nih.gov/). The DNA orthologue searches were conducted using the resident BLAST search engines on either the Harvard site (http://compbio.dfci.harvard.edu/cgi-bin/tgi/Blast/index.cgi) or GenBank site (http://blast.ncbi.nlm.nih.gov/Blast.cgi) (Altschul et al. 1997). If protein sequences were not already available in the database accession, they were generated by first determining the correct reading frame of each Expressed Sequence Tag using the Open Reading Frame (ORF)-finding program at the National Center for Biotechnology Information (http://www.ncbi.nlm.nih.gov/gorf/gorf.html). All putative protein sequences were then “BLASTED” to ensure that they were indeed homologous to the sequence being analyzed— DFR, ANS, F3′H, F3′5′H, or F3GT. Additionally, the five anthocyanin pathway enzymes were also employed to search green, brown, and red algae databases at the Gene Index Project site. As with the other searches, the cDNA of the ORF primary coding sequence was employed.

### Sequence alignments and phylogenetic tree construction

Multiple alignments of DFR, ANS, F3′H, F3GT and F3′5′H DNA and amino acid orthologues were constructed using the CLUSTAL X v1.8 software (Thompson et al. 1997). All alignment settings were employed at default values.

Phylogenetic trees were generated from the genetic distances provided by the CLUSTAL X analysis using the neighbor-joining method (Saitou and Nei 1987) and bootstrap analyses (Felsenstein 1985) consisting of 1,000 replicates were performed. The neighbor-joining trees were visualized with the TREEVIEW program (Page 1996). Bootstrap values less than 500 are not included on cladograms.

We performed additional Maximum Likelihood (ML), Randomized Axelerated Maximum Likelihood (RaxML), Parsimony (MP), and Bayesian (Maximum Clade Credibility) analyses of the DNA and protein alignments using PhyML 3.0 (Guindon et al. 2010), Trex (Stamatakis 2006), MEGA 5.05 (Tamura et al. 2011), and BEAST (Drummond et al. 2012), respectively. These additional analyses were performed using default settings.

## Results and discussion

### Analysis of DFR family members

**Figure 2:**
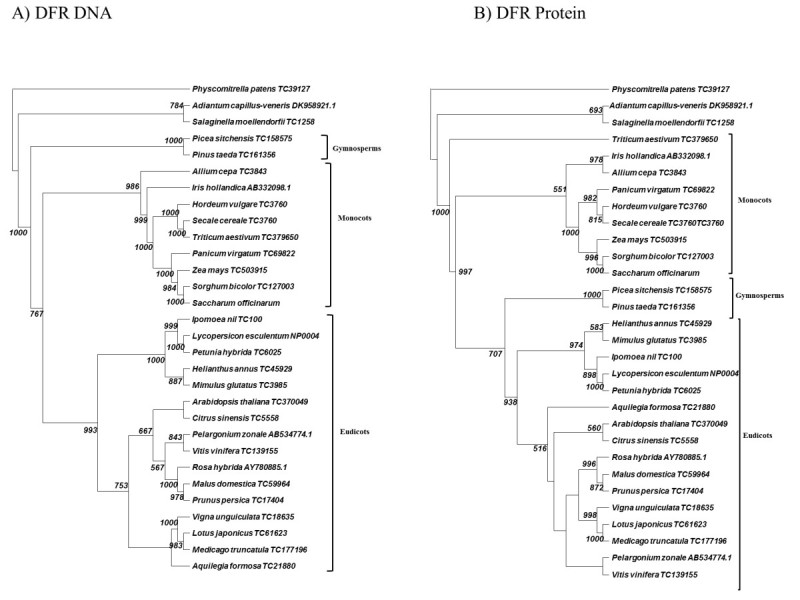
**Cladogram of neighbor-joining analysis for DFR. A)** DNA sequence analysis. **B)** Protein sequence analysis. 1000 bootstraps were performed on each analysis. Bootstrap values less than 500 are excluded.

Dihydroflavonol 4-reductase appears to be one of the most conserved enzymes in the evolution of terrestrial plants. Our DNA cladogram (Figure 2A) shows evidence that this enzyme has representatives with high similarity in all land plants going back to the Bryophytes. The cladogram is rooted with the DFR of the moss *Physcomitrella*, since this is the most ancient ancestor in the analysis. Fern (*Adiantum capillus-veneris*) and spike moss (*Salaginella moellendorfii*) also clade in an outgroup on their own branch. The gymnosperm branch (spruce and pine) lies just below the fern (Figure 2A) with another high bootstrap value (1000). After these outgroups at the base of the tree, the angiosperms branch off into their separate clades of monocotyledons and eudicotyledons, clearly separated from each other.

The DFR protein phylogenetic analysis (Figure 2B) generally follows the same pattern of the DNA cladogram with some anomalies. Again moss roots the protein tree with fern on a neighboring outgroup branch. And again, angiosperms are clearly delineated into eudicots and monocots. The major anomaly arises from the gymnosperm branch clading down near the eudicots with a high bootstrap value (707).

Despite the important phylogenetic relationships that our data suggest, we should be sanguine, but cautious with all these orthologue analyses. Our assumptions throughout this paper are that sequence orthology is the equivalent of functional orthology. Although the homology between sequences is present without doubt, many of these enzymes have not been characterized for functionality. In short, they may be highly similar sequences, but not necessarily functional analogues.

### Analysis of F3GT and ANS family members

**Figure 3:**
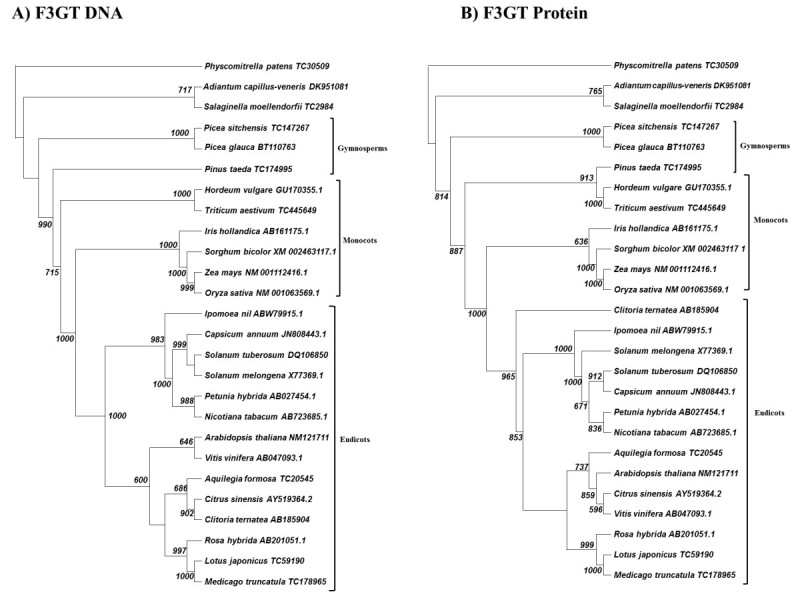
**Cladogram of neighbor-joining analysis for F3GT. A)** DNA sequence analysis. **B)** Protein sequence analysis. 1000 bootstraps were performed on each analysis. Bootstrap values less than 500 are excluded.

**Figure 4:**
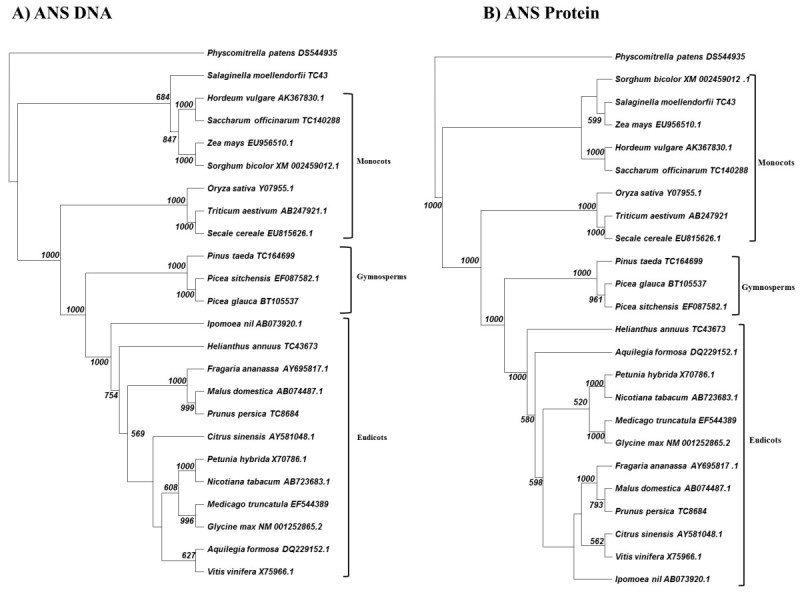
**Cladogram of neighbor-joining analysis for ANS. A)** DNA sequence analysis. **B)** Protein sequence analysis. 1000 bootstraps were performed on each analysis. Bootstrap values less than 500 are excluded.

Both F3GT and ANS demonstrate great conservation, being detectable in the most ancient of plant species (Figures 3, 4). The F3GT DNA cladogram (Figure 3A) demonstrates a straight-forward chronological adherence to proposed phylogeny, while its protein tree shows a minor anomaly (Figure 3B). The anomaly shifts the putative *Pinus taeda* F3GT from an outgroup position and clades it with barley and wheat.

The ANS analysis shows the greatest anomalies of any of the members of the anthocyanin synthesis pathway. Although, the DNA and protein cladograms (Figure 4) show general agreement with known plant phylogeny--with monocots/eudicots and gymnosperms being in separate clades-- we still see the gymnosperm clade being displaced into the center of the clade and not acting as a general outgroup to all angiosperms. Additionally, *Salaginella* ANS becomes part of the monocot clade in both its DNA and protein form. We were not able to locate an ANS orthologue for a representative “true” fern. We attribute this result to the present lack of comprehensive sequencing of DNA in the Pteridaceae genomes.

### Analysis of F3′H and F3′5′H family members

**Figure 5:**
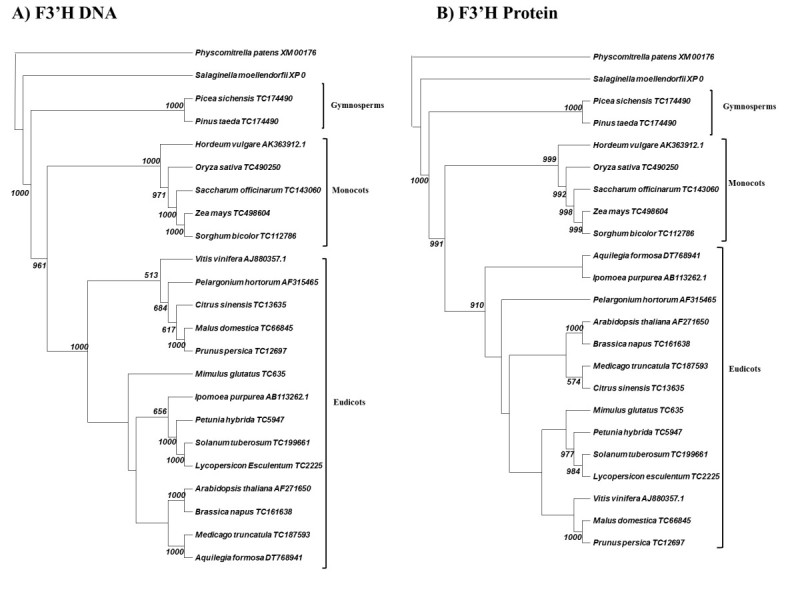
**Cladogram of neighbor-joining analysis for F3′H. A)** DNA sequence analysis. **B)** Protein sequence analysis. 1000 bootstraps were performed on each analysis. Bootstrap values less than 500 are excluded.

The flavonol 3′hydroxylase DNA/protein cladograms follow the same phylogenetic model, and the pattern agrees with our current understanding of plant evolution (Figure 5). Again, moss acts as the outgroup followed by spike moss, gymnosperms, and angiosperms. This result supports the hypothesis that production of cyanidin has been evolutionarily conserved from the bryophytes until modern flowering plants. The resultant branch separation also suggests that there have been negligible structure/function modifications in the F3′H during the major evolutionary shifts from mosses to angiosperms. Again, we could discover no F3′H family members in the limited fern genome database found in GenBank.

Note that flavonol 3′hydroxylase (Figure 5) is one of the loci in our study to show a) no changes between the DNA and protein phylogenies and b) “proper” phylogeny in the protein cladogram. This result suggests a strong positive selection pressure to ensure that F3′H DNA and protein structure drifted little over evolutionary time.

**Figure 6:**
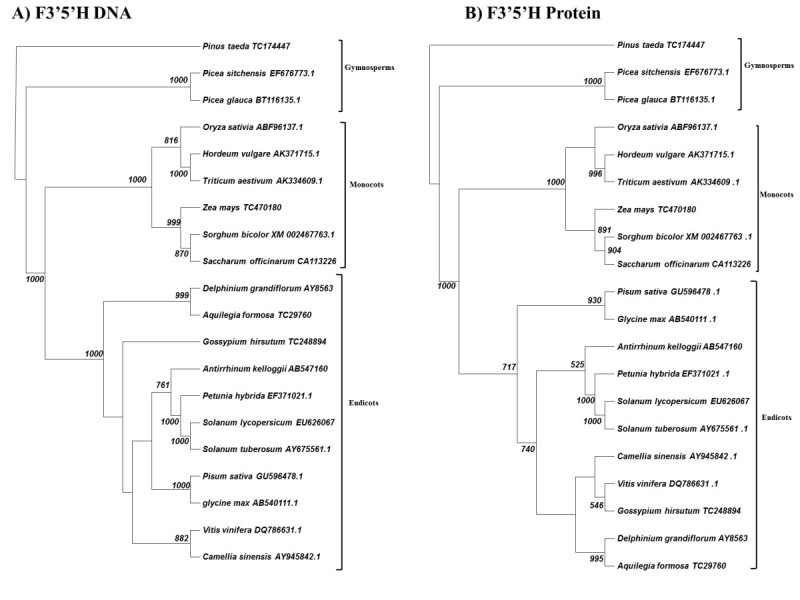
**Cladogram of neighbor-joining analysis for F3′5′H. A)** DNA sequence analysis. **B)** Protein sequence analysis. 1000 bootstraps were performed on each analysis. Bootstrap values less than 500 are excluded.

The flavonol 3′5′hydroxylase is the most recent addition to the anthocyanin synthesis pathway. We found that F3′5′H cannot be traced to earlier antecedents than gymnosperms (Figure 6). Spike mosses, ferns and mosses show no evidence of this enzyme, which is necessary for the production of the purple/blue anthocyanin pigment delphinidin. As with the F3′H, the F3′5′H family demonstrates no changes between the DNA and protein phylogenies.

### Ancient evolution in the anthocyanin pathway

The most ancient terrestrial plants, the bryophytes, arose 450–425 mya when the ozone layer started to form over the earth (Duff and Nickrent 1998; Shear 2000). Before that time, only algae were extant. Algae developed a range of UV-absorbing compounds, since they still needed to be shielded from ultraviolet light even in the water (Rozema et al. 2002; Xue et al. 2005), but they did not evolve anthocyanins. Since the UV-B wavelengths of light were attenuated by the water column for algae, Rozema et al. (2002) suggests that it is likely that phenolic pigments evolved in terrestrial plants to protect them from increased levels of UV-B found on land. Additionally, the Siluro-Devonian colonization of the land by plants was possible in part because the new shielding properties of the ozone layer arose concurrently with the evolution of endogenous color pigments to protect plants from UV light not filtered by the upper atmosphere (Duff and Nickrent 1998; Shear 2000; Rozema et al. 2002).

We have found that mosses, whose ancestors were the first land plants, may have made anthocyanins as early as 450 mya. Although we do not know if the enzymes were functionally orthologous to those homologues from more recently evolved plant species, the anthocyanin pathways were apparently available as long ago as the late Ordovician period to make both pelargonidins and cyanidins (Figures 2, 3, 4, 5). Our result is supported by known evolutionary biochemistry data (Mues 2002; Rausher 2006). Since flowering plants would not evolve for hundreds of millions of years after mosses, we can only assume that a primary function of these ancient pigments in bryophytes was UV protective and antioxidant in nature. This conclusion is supported in the moss literature (Bendz 1961; Post 1990; Mues 2002; Dunn and Robinson 2006). Although Bendz (1961) found the flavone Luteolin in some moss species, other species such as *Sphagnum capillifolium* have been shown to produce brick-red cyanidin (Mues 2002), especially under environmentally stressed conditions (Steyn et al. 2002; Bonnett et al. 2010).

Wolf et al. (2010) could not detect the presence of anthocyanin pigments in stressed *Physcomitrella patens*. However, other moss species readily produce detectable quantities of anthocyanin pigment after stress. Steyn et al. (2002) found that anthocyanins generally accumulate in *S. capillifolium* peripheral tissues exposed to high irradiance or on occasion in the shade due to a disparity between light capture, carbon dioxide incorporation and carbohydrate consumption. Pigment analysis of red arctic moss under UV stress, due to ozone depletion, has shown increases in anthocyanin pigment and decreases in chlorophyll concentrations, largely accounting for the visible alteration in these mosses from green to red (Post 1990). Post (1990) proposed that these changes in pigmentation are consistent with photo-protection, and they are linked to light dependent variations in plastid structure. Dunn and Robinson (2006) observed similarly that “cosmopolitan” mosses found in more temperate regions consistently had reduced levels of anthocyanins in comparison to their arctic cognates. Although it appears that production of these photo-protective pigments is a new, useful adaptation for the bright, UV-rich arctic environment, it is more likely we are looking at a primordial atavistic reaction to the increase in damaging UV fluence.

There is evidence that non-anthocyanin flavonoids, such as the quercetin-glycosides (Herrmann 2006), arose before anthocyanins as a safeguard against UV (Markham 1988; Stafford 1991; Rozema et al. 2002), but that does not make anthocyanins any less important in their own evolutionary pathway and purpose. Stafford (1991) suggested that initially flavonoids, including anthocyanins, may not have been UV protective because they were probably being produced at low concentrations, and further that flavonoids may have initially been primarily phytohormonal in their function. If this is the case, then we may assume that evolutionary selection, beginning in moss, may have eventually allowed enough accumulation of flavonoids to allow them to assist in the UV defensive role (Stafford 1991). Although phenylpropanoid phenolics and sinapic acid esters (Li et al. 1993) could also serve as UV filters and may have been the initial ones, their absorption coefficients are lower than flavonoids. Plants producing large quantities of anthocyanins for UV protection would not have evolved if pigments such as sinapic esters were a sufficient defense.

Given that the anthocyanin pathway appears so conserved, we examined databases of “lower” plants to determine how far back in evolutionary time these pigments can be traced. However, a search of the brown (Ectocarpus), red (*Porphyra*), and green (Volvox) algae databases in GenBank and the Gene Index manifested no homologous sequences to any enzymes in the anthocyanin production pathway (data not shown). Again, the work of Caputi et al. (2012) does indicate a UGT being present in *Chlamydomonas*, but this transferase is not part of the UGT clade of anthocyanins. These results agree with known algal biochemistry (Rausher 2006). Although our lack of success in finding homologous sequences may be due to limitations in the sequence databases of these lower photosynthetic organisms, it seems equally likely that the final pathway components simply did not exist until land plants evolved.

We were able to demonstrate that ferns apparently conserved at least two of the major enzymes in the anthocyanin pathways in DFR and F3GT (Figures 2, 3), while spike mosses retained all the major enzymes (Figures 2, 3, 4, 5). We do not believe that ferns are lacking in the ANS and F3′H loci, since more ancient plant species have these genes, however more comprehensive genome databases are required to probe into which loci are actually present in the fern genome.

When ferns evolved ~400 mya (Pryer et al. 2004), they faced the same photo-stresses as their more ancient counterparts, so selection pressure to keep anthocyanins was still present to ensure continued protection. Additionally, anthocyanins can act as chemical deterrents of herbivory, which would have become an important survival issue in ferns as larger animals evolved (Rausher 2006).

### More “recent” evolution in the anthocyanin pathway

Gymnosperms appear to be the first land plants (300–325 mya) [19] in which we can observe the presence of the F3′5′H enzyme, which is required to make delphinidin. We cannot detect this hydroxylase expressed in moss, spike moss, or fern (data not shown). It is not entirely clear why this purple blue pigment arose in gymnosperms, but we can hypothesize that it may have been related to the greater spread of plants to higher elevations during the Carboniferous Era. Delphinidin has greater absorption in the red part of the spectrum (maximum 557 nm) and a greater reflection in the UVB portion of the spectrum than either pelargonidin or cyanidin (Harborne 1958). This observation would suggest that as gymnosperms grew taller and migrated to higher elevations, there would be selection for a pigment, i.e. delphinidin, with greater protection against higher fluence of UV light. Alternatively, there is some evidence that gymnosperms may have been the first plants to use blue pigment to attract foraging animals to distribute seeds. Even today some gymnosperms such as the juniper still produce blue, “berry-like” structures that are actually the female seed cone of the juniper tree (Salomonson 1978).

Angiosperms arose during the late Triassic Era (170–245 mya) (Moore et al. 2007), and the five major genes in the anthocyanin pathways continued to be conserved, with the added ability to produce delphinidin being passed down to flowering plants with the F3′5′H enzyme.

Monocots diverged from eudicots 75–100 million years ago (Kellogg 2001). As the monocots diverged, our data suggest that the components of the anthocyanin production pathway, although conserved functionally, began to show greater evidence of amino acid divergence. Except for slight variations, this conclusion seems to be particularly accurate with analyses of DFR, F3′H, F3′5′H, and F3GT (Figures 2, 3, 5, 6). These gene products demonstrate clear phylogenetic divisions between monocots and eudicots. Despite the structural variations in these enzymes, there were no chemical changes in the major anthocyanin pigments (pelargonidin, cyanidin, and delphinidin) after the monocot/eudicot split, even though we eventually see more anthocyanin variants evolve beyond the major ones (Grotewold 2006).

The ANS family of orthologues seems to demonstrate a different divergence pattern from the rest of the anthocyanin synthesis pathway components (Figure 4). The biggest anomaly is that the gymnosperm ANS appear as an outgroup to the eudicots and not closer to the root of the tree. This ANS clading seems to reflect a general lack of divergence.

As another example of this lack of divergence, we see a grouping of monocot grasses clading with the fern ANS (Figure 4A,B). Since those grasses evolved long after the ferns and gymnosperms, we are likely seeing either a) independent evolution leading those monocots back to a higher orthology with an ancient ancestor (Stafford 1991), or b) evidence that in certain plant lineages there were few changes in ANS structure from fern through these grasses. This selection may have arisen in response to grasses being exposed in open fields to higher levels of UV than other plants, which evolved in potentially more shaded environments.

## Conclusions

Our data support a hypothesis that has been suggested in the literature over the last decade (Seitz et al. 2006; Halbwirth 2010). This hypothesis is that F3′5′H is clearly a component of several coniferous genomes and so these blue pigments emerged before the angiosperm/gymnosperm divergence. Although it may not necessarily be accurate that these pigments were first used for reproductive purposes in ancient gymnosperms, they were clearly present in these genomes.

Further, cyanidin has been considered “the most primitive” anthocyanin for a long time because it is the most common anthocyanin in gymnosperms and almost ubiquitous in leaves and stems (Mueller and Walbot 2001; Halbwirth 2010). We do not dispute this conclusion. In fact, our data support that cyanidin production is even more ancient than gymnosperms because the enzymatic components that produce cyanidin are present in mosses. The high conservation of the F3′H phylogeny (Figure 5) from DNA to protein supports the significance of cyanidin production.

One final observation is that the DNA and protein cladograms for DFR and ANS (Figure 2, 4) do not mirror each other. Differences between DNA and protein phylogenies often arise from silent DNA mutations, which ensure protein sequence conservation, but produce phylogenetic divergence at the level of DNA. However, our data are suggesting something more complex. We are not seeing evidence in DFR and ANS that DNA sequences are being selected and driven by silent mutations because such changes would inevitably be observed as alterations in DNA-based phylogeny with a continued preservation of the protein sequences. Instead, the DNA sequences are conserved and changes occur in the protein sequences. How can we explain this?

To ensure no data errors, we double-checked, using BLAST, that all protein sequences employed in our analyses were appropriately homologues and being translated from the correct open reading frame.

We also employed “Multiple Alignment Using Fast Fourier Transform” (Katoh and Kuma 2002) as an alternative method of alignment, but found no difference in our findings from implementing the “classic” methods of alignment (data not shown).

Finally, we determined whether our results are not just a consequence of the neighbor-joining (NJ) analysis. We performed additional ML, RaxML, Parsimony, and Bayesian analyses of the DNA and protein alignments using PhyML 3.0 (Guindon et al. 2010), Trex (Stamatakis 2006), MEGA 5.05 (Tamura et al. 2011), and BEAST (Drummond et al. 2012), respectively. No substantive variance could be seen in the ML, RaxML, MP, or Bayesian versus NJ trees constructed (data not shown). These controls support our conclusion that our phylogenetic results are not simply based on a biased analysis.

One hypothesis for the DNA/protein cladogram divergences is that two separate and distinct selection processes are occurring. One is a DNA selection process that tolerates as few changes as possible in the primary coding sequence and where selection is being driven by divergence and drift. The second selection process would allow minor mutations to occur in the DNA coding sequence, leading to amino acid selection and functional maintenance of the proteins under fluctuating environmental conditions. A minor change could occur in the DNA sequence as a small percentage of the total length. That minor change would be amplified in the amino acid sequence of the protein because it would turn into a larger percentage of the total protein length. This phenomenon would lead to what we are observing: i.e. the DNA sequence highly conserved with the protein being selected for functionality and allowing minor null or silent mutations to encroach.

## Authors’ original submitted files for images

Below are the links to the authors’ original submitted files for images. Authors’ original file for figure 1 Authors’ original file for figure 2 Authors’ original file for figure 3 Authors’ original file for figure 4 Authors’ original file for figure 5 Authors’ original file for figure 6
