# Supplementary figures and images for: A phylogenetic examination of the primary anthocyanin production pathway of the Plantae
Source: Bot Stud. 2014 Jan 25;55:10. doi: 10.1186/1999-3110-55-10 (PMC5432750; doi:10.1186/1999-3110-55-10)

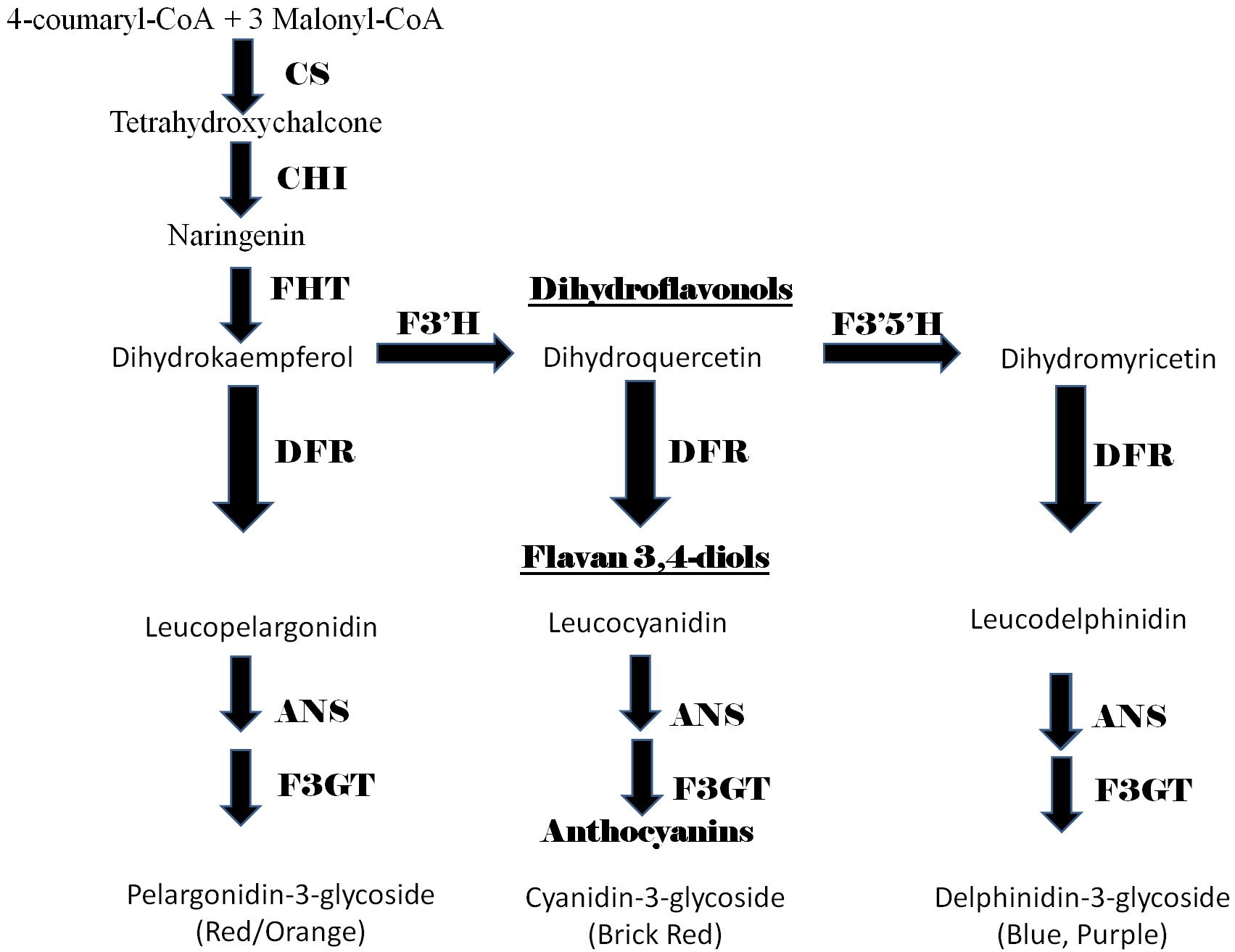

Supplement: Supplementary file 1 — Authors’ original file for figure 1 [file 40529_2013_61_MOESM1_ESM.tiff]

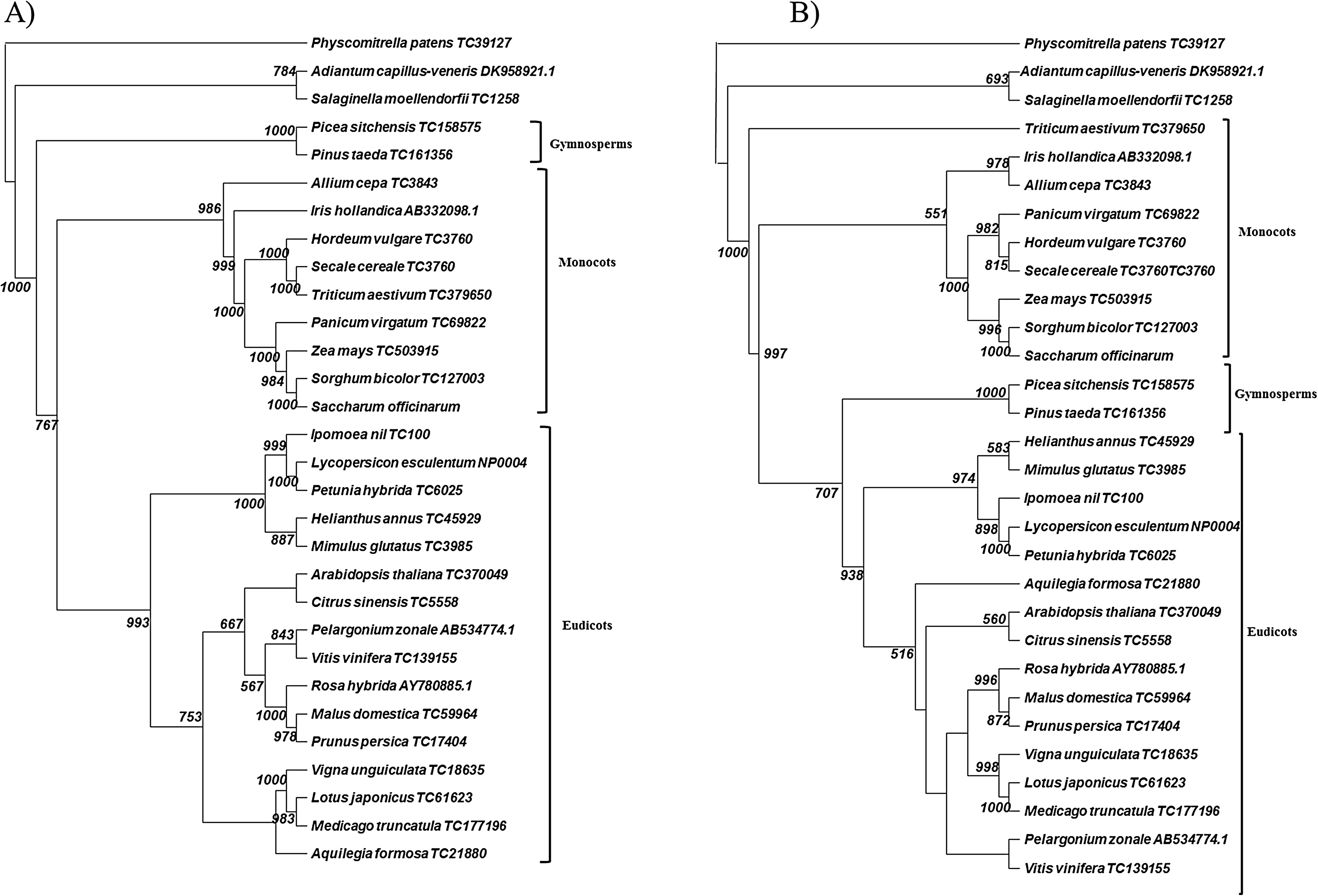

Supplement: Supplementary file 2 — Authors’ original file for figure 2 [file 40529_2013_61_MOESM2_ESM.tiff]

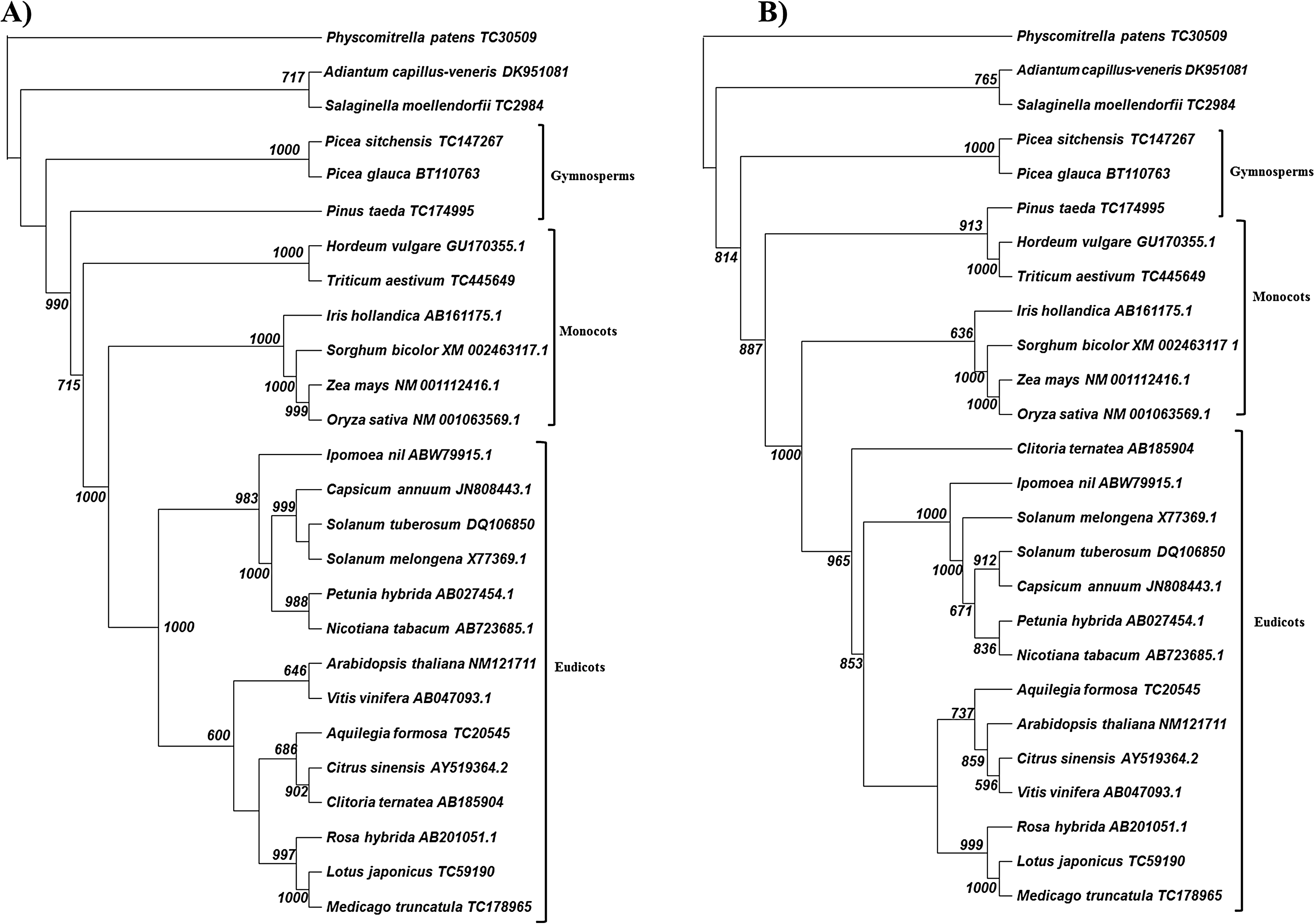

Supplement: Supplementary file 3 — Authors’ original file for figure 3 [file 40529_2013_61_MOESM3_ESM.tiff]

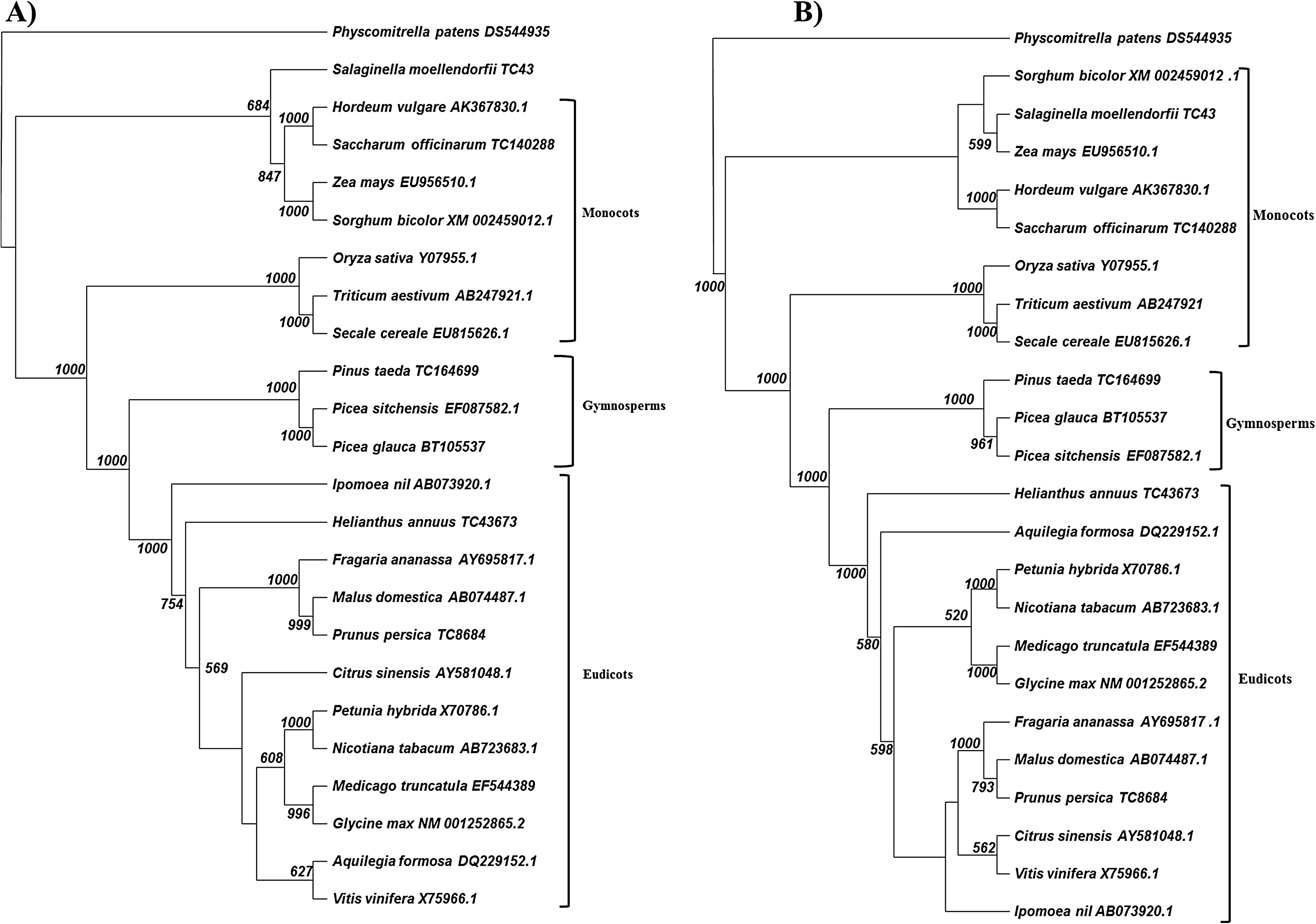

Supplement: Supplementary file 4 — Authors’ original file for figure 4 [file 40529_2013_61_MOESM4_ESM.tiff]

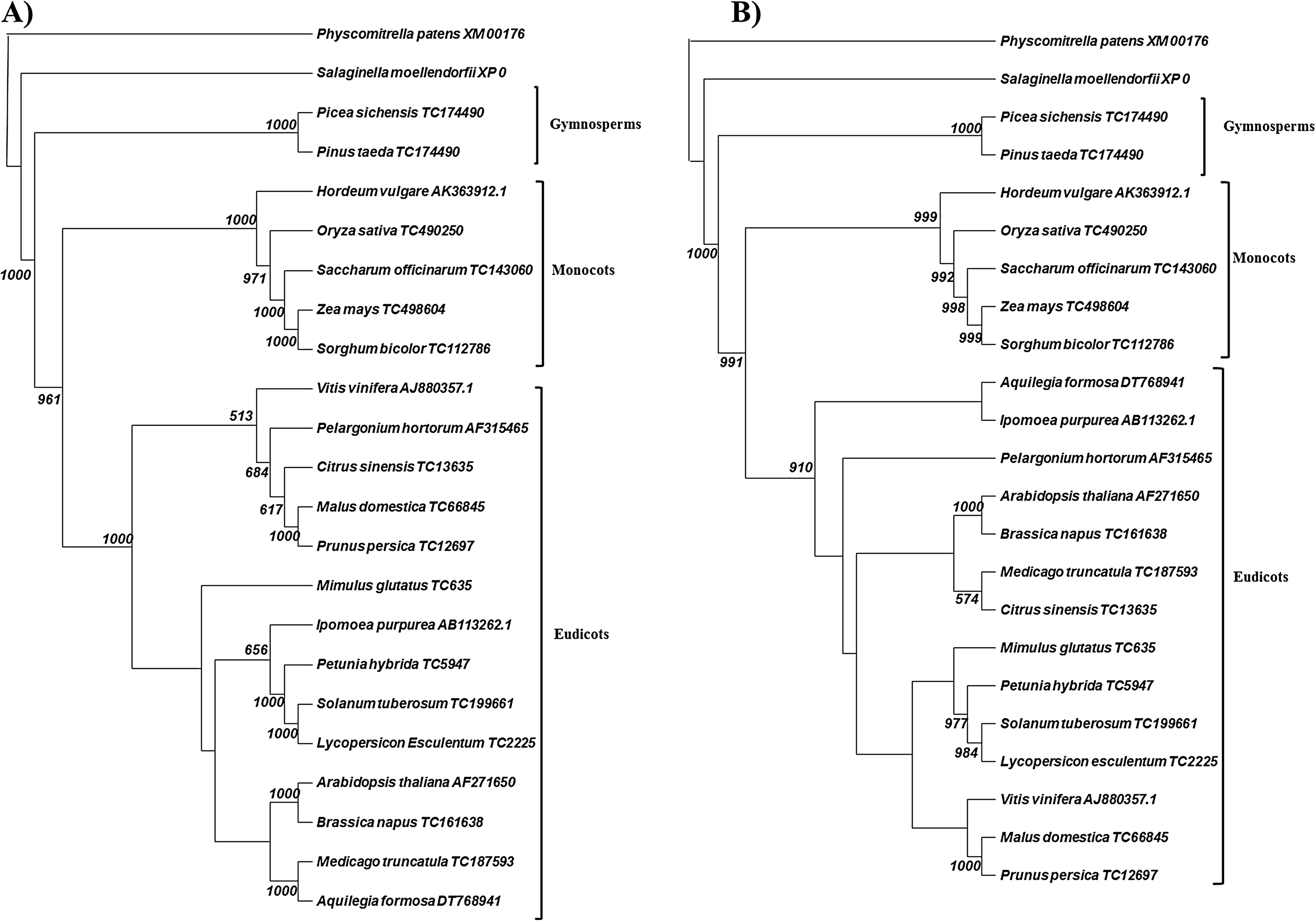

Supplement: Supplementary file 5 — Authors’ original file for figure 5 [file 40529_2013_61_MOESM5_ESM.tiff]

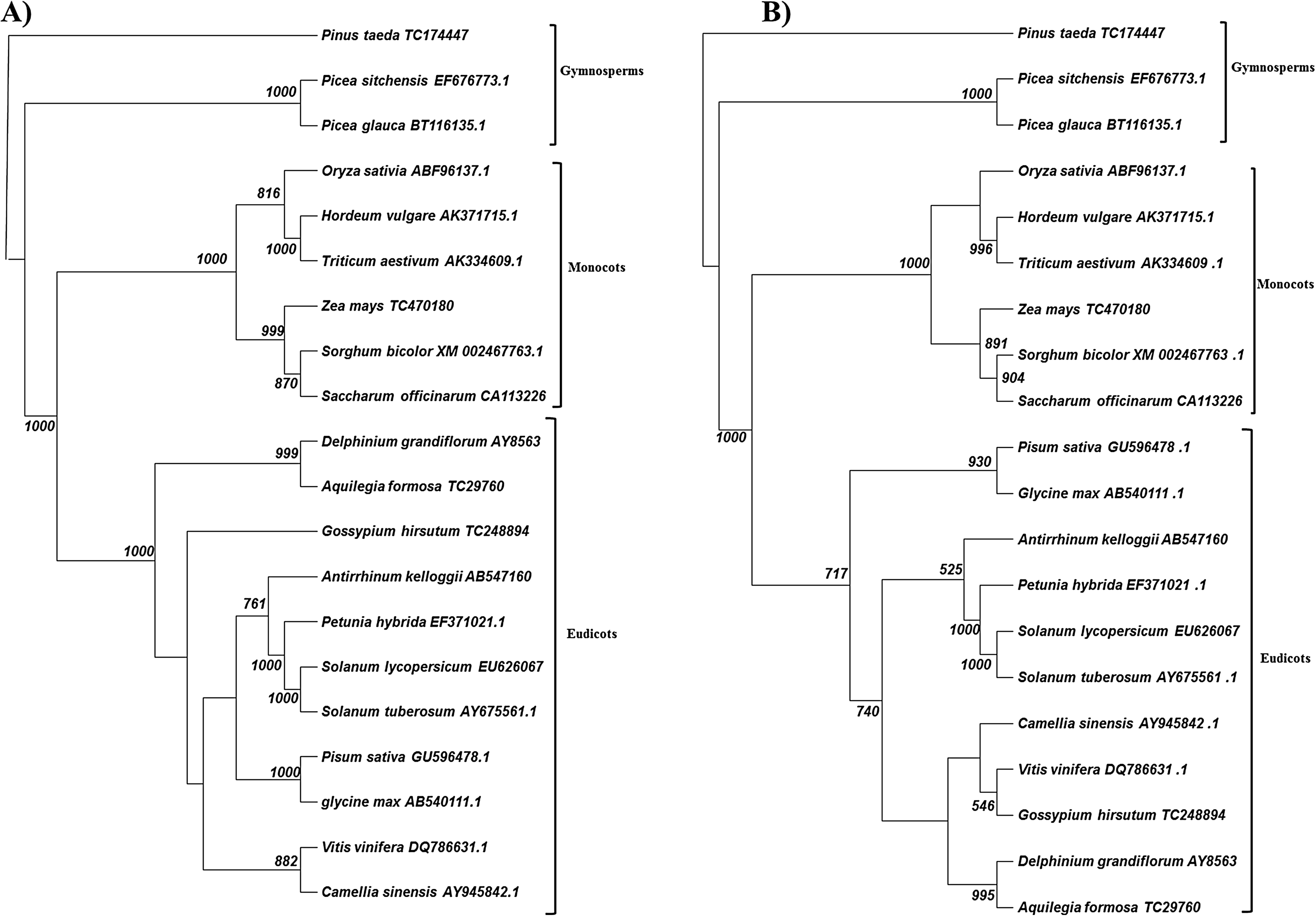

Supplement: Supplementary file 6 — Authors’ original file for figure 6 [file 40529_2013_61_MOESM6_ESM.tiff]
